# The prevalence of the *iutA* and *ibeA* genes in *Escherichia coli* isolates from severe and non-severe patients with bacteremic acute biliary tract infection is significantly different

**DOI:** 10.1186/s13099-021-00429-1

**Published:** 2021-05-18

**Authors:** Mahoko Ikeda, Tatsuya Kobayashi, Fumie Fujimoto, Yuta Okada, Yoshimi Higurashi, Keita Tatsuno, Shu Okugawa, Kyoji Moriya

**Affiliations:** 1 Department of Infection Control and Prevention, The University of Tokyo Hospital, 7-3-1, Hongo, Bunkyo-ku, Tokyo, 113-8655 Japan; 2 Department of Infectious Diseases, The University of Tokyo Hospital, 7-3-1, Hongo, Bunkyo-ku, Tokyo, Japan

**Keywords:** Extraintestinal pathogenic *Escherichia coli*, Biliary tract infection, Bacteremia, Severity, Virulence factors, *iutA*, *ibeA*

## Abstract

**Background:**

Although *Escherichia coli* is the most frequently isolated microorganism in acute biliary tract infections with bacteremia, data regarding its virulence are limited.

**Results:**

Information on cases of bacteremia in acute biliary tract infection in a retrospective study was collected from 2013 to 2015 at a tertiary care hospital in Japan. Factors related to the severity of infection were investigated, including patient background, phylogenetic typing, and virulence factors of *E. coli*, such as adhesion, invasion, toxins, and iron acquisition.

In total, 72 *E. coli* strains were identified in 71 cases, most of which primarily belonged to the B2 phylogroup (68.1%). The presence of the *iutA* gene (77.3% in the non-severe group, 46.4% in the severe group, *P* = 0.011) and the *ibeA* gene (9.1% in the non-severe group, and 35.7% in the severe group, *P* = 0.012) was significantly associated with the severity of infection. Among the patient characteristics, diabetes mellitus with organ involvement and alkaline phosphatase were different in the severe and non-severe groups.

**Conclusions:**

We showed that bacteremic *E. coli* strains from acute biliary tract infections belonged to the virulent (B2) phylogroup. The prevalence of the *iutA* and *ibeA* genes between the two groups of bacteremia severity was significantly different.

## Background

*Escherichia coli* is the most frequently isolated microorganism in acute biliary tract infections [1, 2], and the associated bacteremia is mainly caused by *E. coli* [3]. Biliary tract infections normally start with the stasis of bile flow, and once intestinal bacteria flow into the bile duct, an acute biliary infection can develop. The causative organisms in bile cultures of acute biliary infections were shown to be polymicrobial [3, 4]. These pathogens were considered intestinal commensals of low virulence [5].

*E. coli* can be differentiated depending on its pathogenicity as commensal, which are considered avirulent; intestinal pathogenic; extraintestinal pathogenic, such as uropathogenic, neonate meningitis groups and sepsis-associated groups; and as avian pathogenic [6, 7].

Acute biliary infections, such as cholangitis and cholecystitis, can develop into a severe infection with bacteremia. In cases of severe infection, mortality can reach 10% [8]. This begs the question, if the causative organisms are considered to be avirulent, why did the patients suffer severe disease and even death?

We hypothesized that the causative *E. coli* were virulent, and this influenced the severity of acute biliary infection. Uropathogenic *E. coli* strains, which cause urinary tract infections, have been extensively studied, and have shown to express several virulence factors, such as those involved in adhesion (*afaB/C*, *fimH*, *iha*, *papC*, *papG2*, and *sfaD/E*), toxicity (*cnf1*, *hlyA*, *sat*, and *usp*), iron acquisition (*fyuA*, *ironN*, *iucD* and *iutA*), capsule synthesis (*kpsMT2*), and miscellaneous genes (*cvaC*, *ibeA*, *ompT*, *tcpC*, and *traT*). These virulence factors play important roles at each step of infection [9]. Studies on *E. coli* strains from bacteremia in biliary tract infections are however limited. A previous study showed different prevalences of 10 virulence factors (*afa*, *cnf1*, *fimH*, *foc*, *hlyA*, *iutA*, *papG* class I, *papG* class II, *papG* class III, and *sfa*) in Biliary pathogenic *E. coli *(BEC) strains compared in *E. coli* strains from blood cultures of patients with acute urinary tract infections [10]. BEC strains might exist and have unique traits as uropathogenic *E. coli* strains have.

Pathogenic *E. coli* express many common virulence factors even at different sites of infection, but an organ-specific strategy is needed to identify specific virulence factors. These common and/or different traits are now studied rigorously to understand pathogenesis and to cope with infection at different foci.

Our study aimed to analyze the relationship between the severity of biliary tract infections and the prevalence of the virulence factors of BEC isolates.

## Results

A total of 71 cases of bacteremic acute biliary tract infections (72 BEC isolates) were identified. In one of the patients, two *E. coli* isolates with different colony morphology were detected; we have included both isolates in the study.

The patients were grouped depending on their severity level, and the characteristics of the severe group (Pitt’s score ≥ 2) and the non-severe (score < 2) group were compared. Patient background, such as age, gender, and comorbidity were the same in the two groups, except for diabetes mellitus (DM) with organ involvement (Table 1). More than half of the patients in both groups presented abnormalities of the biliary tract and had experienced biliary tract infections in the past.

**Table 1:** Comparison of patient backgrounds and symptoms, and severity of bacteremia

	Total	Non-severe group (Pitt < 2)	Severe group (Pitt ≥ 2)	*P* value
Number of patients	71	44	27	
Age (median, range)	75 (31–94)	72.9 (34–93)	72 (31–94)	0.38
Gender (male: female)	F28 M43	F16 M28	F12 M15	0.618
Nursing home	4	2	2	0.632
Antibiotic use within 3 months	29	19	10	0.479
Charlson index (median, range)	4 (0–11)	4 (0–11)	3 (0–8)	0.104
Collagen diseases	4	4	0	0.29
Diabetes mellitus without organ involvement	7	3	4	0.415
Diabetes mellitus with organ involvement	20	17	3	0.015*
Malignancy	49	31	18	0.613
Abnormality of biliary tract	43	29 (65.9%)	14 (52%)	0.318
Abnormality of gastrointestinal tract	38	25	13	0.625
Previous history of acute biliary infection	33	21	12	0.811
Previous history of bacteremia	11	7	4	1
Duration between onset of symptoms and admission (days)	0 (− 24 to 10)	0 (− 24 to 4)	0 (−10 to 10)	0.924
Fever	39	23	16	0.629
Immunosuppressant use	27	19	8	0.318
Artificial device in biliary tract	19	11	8	0.784

^*^With statistical significance

The laboratory data showed that the liver enzymes and biliary markers analyzed were elevated to the same degree in the two severe and non-severe groups, except for alkaline phosphatase (ALP), and the median white blood cell counts, which were higher in the non-severe group (Table 2).

**Table 2:** Comparison of laboratory data and severity of bacteremia

	Total	Non-severe group (Pitt < 2)	Severe group (Pitt ≥ 2)	*P* value
White blood cell counts (per μL) median (min–max)	9800 (1200–25,600)	10,500 (2400–25,600)	8600 (1200–23,200)	0.0492*
Platelets (× 10,000/μL)	16.1 (7.0–38.1)	17.6 (7–38.1)	15.2 (7.0–35.1)	0.211
Albumin (g/dL)	3.2 (1.0–4.5)	3.15 (1.0–4.5)	3.5 (2.0–4.2)	0.136
Total bilirubin (mg/dL)	2.0 (0.4–11.6)	2.0 (0.4–11.6)	2.2 (0.5–7.0)	0.148
AST (U/L)	100 (16–1591)	98 (16–1026)	133 (17–1591)	0.152
ALT (U/L)	91 (11–724)	77 (13–569)	100 (11–724)	0.4
g-GTP (U/L)	328 (11–2118)	346 (11–2118)	288 (14–817)	0.621
ALP (U/L)	733 (143–3520)	855 (143–3520)	594(232–3278)	0.026^*^
CRP (mg/dL)	3.76 (0.08–22.32)	3.48 (0.08–18.53)	5.0 (0.11–22.32)	0.687

*AST* aspartate aminotransferase, *ALT* alanine aminotransferase, *g-GTP* gamma-glutamyl transpeptidase, *ALP* alkaline phosphatase, *CRP* C-reactive protein

^*^With statistical significance

Next, we compared the phylogenetic groups, ST lineages, and virulence factors of BEC by the severity level (Table 3). The most detected phylogenetic group was B2 in both groups (70.5% in non-severe, 64.3% in severe), and the proportion of each phylogenetic group between the severe and non-severe groups was similar. BEC isolates mainly belonged to the B2 phylogenetic group (68.1%), and to ST131 (23.6%) and the ST95 (19.4%) lineages, as detected by multi-locus sequencing typing (MLST). The extended-spectrum-beta-lactamase (ESBL)-producing isolates were detected 12 isolates, including 7 of ST131 lineage, and others were one each of ST10, ST95, ST354, ST648, and ST1196 lineages, respectively. The proportions of STs and prevalence of the ESBL-producing isolates were not significantly different between the two groups.

**Table 3:** Comparison of patterns of phylogenetic groups and prevalence of virulence factors and severity of bacteremia

	Total	Non-severe group (Pitt < 2)	Severe group (Pitt ≥ 2)	*P* value
Phylogenetic group	72	44	28	0.732
B2	49 (68.1%)	31 (70.5%)	18 (64.3%)	
B1	8 (11.1%)	3 (6.8%)	5 (17.9%)	
E	6 (8.3%)	4 (9.1%)	2 (7.1%)	
A	4 (5.6%)	2 (4.5%)	2 (7.1%)	
F	4 (5.6%)	3 (6.8%)	1 (3.6%)	
D	1 (1.4%)	1 (2.2%)	0	
ST				0.165
ST131	17 (23.6%)	13 (29.5%)	4 (14.3%)	
ST95	14 (19.4%)	8 (18.2%)	6 (21.4%)	
ST73	5 (6.9%)	4 (9.1%)	1 (3.6%)	
ST127	2 (2.8%)	2 (4.5%)	0	
ST405	2 (2.8%)	2 (4.5%)	0	
ST357	2 (2.8%)	0	2 (7.1%)	
ST453	2 (2.8%)	1 (2.2%)	1 (3.6%)	
ST1196	2 (2.8%)	1 (2.2%)	1 (3.6%)	
ST10	1 (1.4%)	1 (2.2%)	0	
ST117	1 (1.4%)	1 (2.2%)	0	
ST126	1 (1.4%)	1 (2.2%)	0	
ST144	1 (1.4%)	1 (2.2%)	0	
ST345	1 (1.4%)	1 (2.2%)	0	
ST354	1 (1.4%)	0	1 (3.6%)	
ST420	1 (1.4%)	1 (2.2%)	0	
ST429	1 (1.4%)	1 (2.2%)	0	
ST457	1 (1.4%)	1 (2.2%)	0	
ST607	1 (1.4%)	0	1 (3.6%)	
ST648	1 (1.4%)	1 (2.2%)	0	
ST683	1 (1.4%)	0	1 (3.6%)	
ST1193	1 (1.4%)	0	1 (3.6%)	
ST1304	1 (1.4%)	0	1 (3.6%)	
ST1380	1 (1.4%)	1 (2.2%)	0	
ST1415	1 (1.4%)	1 (2.2%)	0	
ST1851	1 (1.4%)	0	1 (3.6%)	
ST2074	1 (1.4%)	1 (2.2%)	0	
ST2732	1 (1.4%)	0	1 (3.6%)	
ST3532	1 (1.4%)	0	1 (3.6%)	
ST6998	1 (1.4%)	0	1 (3.6%)	
ST7344	1 (1.4%)	0	1 (3.6%)	
Un-typable	4 (5.6%)	1 (2.2%)	3 (10.7%)	

The presence of the *iutA* gene (77.3% in the non-severe group, 46.4% in the severe group, *P* = 0.011) and the *ibeA* gene (9.1% in non-severe group, and 35.7% in severe group, *P* = 0.012) were significantly associated with severity as determined by univariable analysis (Table 4), whereas patient characteristics were not significantly associated with severity.

**Table 4:** Comparison of prevalence of virulence factor-encoding genes and severity of bacteremia

	Total	Non-severe group (Pitt < 2)	Severe group (Pitt ≥ 2)	*P* value
Adhesion				
* papC*	21	15	6	0.296
* papG2*	10	8	2	0.297
* sfaD/E*	8	4	4	0.703
* fimH*	69	42	27	1
* afaB/C*	2	1	1	1
* iha*	23	16	7	0.438
Toxin				
* usp*	56	35	21	0.773
* cnf1*	9	5	4	0.728
* hlyA*	9	5	4	0.728
* sat*	22	16	6	0.202
Iron uptake				
* fyuA*	52	35	17	0.108
* iroN*	16	10	6	1
* iucD*	24	18	6	0.124
* iutA*	47	34	13	0.011*
Capsule				
* kpsMT2*	51	33	18	0.427
Miscellaneous				
* ibeA*	14	4	10	0.012*
* traT*	49	33	16	0.128
* cvaC*	6	6	0	0.753
* ompT*	6	6	0	0.075
* TcpC*	12	9	3	0.346

^*^With statistical significance

The distribution of the *iutA* and the *ibeA* genes according to sequence types is shown in Table 5. Only one isolate (ST429) was both *iutA* and *ibeA*-positive. The ST131 (n = 17) and the ST73 (n = 5) isolates were all *iutA*-positive. Among the *ibeA*-positive isolates (n = 14), ST95 accounted for 35.7%.

**Table 5:** Distribution of the *iutA* and the *ibeA* genes according to sequence types

	Total	*iutA* + *ibeA* +	*iutA* + *ibeA −*	*iutA −* *ibeA* +	*iutA –* *ibeA −*
ST131	17	0	17 (100%)	0	0
ST95	14	0	6 (42.9%)	5 (35.7%)	3 (21.4%)
ST73	5	0	5 (100%)	0	0
ST127	2	0	1 (50%)	0	1 (50%)
ST405	2	0	1 (50%)	0	1 (50%)
ST357	2	0	0	2 (100%)	0
ST453	2	0	1 (50%)	0	1 (50%)
ST1196	2	0	2 (100%)	0	0
ST10	1	0	1 (100%)	0	0
ST117	1	0	1 (100%)	0	0
ST126	1	0	0	1 (100%)	0
ST144	1	0	1 (100%)	0	0
ST345	1	0	1 (100%)	0	0
ST354	1	0	0	1 (100%)	0
ST420	1	0	0	1 (100%)	0
ST429	1	1 (100%)	0	0	0
ST457	1	0	1 (100%)	0	0
ST607	1	0	1 (100%)	0	0
ST648	1	0	1 (100%)	0	0
ST683	1	0	1 (100%)	0	0
ST1193	1	0	1 (100%)	0	0
ST1304	1	0	0	0	1 (100%)
ST1380	1	0	0	0	1 (100%)
ST1415	1	0	0	0	1 (100%)
ST1851	1	0	0	1 (100%)	0
ST2074	1	0	1 (100%)	0	0
ST2732	1	0	1 (100%)	0	0
ST3532	1	0	0	0	1 (100%)
ST6998	1	0	0	1 (100%)	0
ST7344	1	0	0	0	1 (100%)
Un-typable	4	0	2 (50%)	1 (25%)	1 (25%)
Total	72	1	46	13	12

## Discussion

We found that factors as DM with organ involvement and values of ALP were different in the two groups analyzed. Hyperglycemia due to DM, which is a metabolic disorder, impairs leucocyte functions [11, 12], and it has been shown that DM is related to the severity or mortality of sepsis [13, 14]. However, opposite results have also been reported, for example, septic patients with DM develop acute respiratory failure with less frequency [15], and DM is not associated with the mortality of *Enterobacterales* bacteremia [16]. Importantly, DM without complications was associated with the 30-days mortality caused by *Staphylococcus aureus* bacteremia, while DM with complications was not [17]. Overall, the mechanism and effect on the severity of bacteremia are not clear, and a more detailed analysis on DM and the immune response in bacteremia would be needed.

ALP levels, which are related with cholestasis, are elevated in acute biliary tract infection and in other diseases such as primary biliary cholangitis, primary sclerosing cholangitis, and non-hepatobiliary disease, in a clinical setting [18]. ALP was measured according to the method of the Japan Society of Clinical Chemistry [19], which differ from that of the International Federation of Clinical Chemistry and Laboratory Medicine. The Japanese method is influenced by ALP derived from the small intestine, and therefore the ALP values observed in the severe and non-severe might be influenced by this, affecting the accuracy of the determination. Here, the values of each ALP fractions were not measured due to the retrospective characteristics of the study.

Next, we found BEC mainly belonged to the B2 phylogroup, the most frequent group among extra-intestinal pathogenic *E. coli* [6]. The sequence types of the isolates were diverse; however, no differences were found between the non-severe and severe groups.

Acute biliary tract infections are caused by obstruction/stasis of biliary flow and influx of intestinal microorganisms. The human intestinal tract has been recognized as a reservoir of extraintestinal pathogenic *E. coli* strains, such as uropathogenic *E. coli* [20]. Once *E. coli* translocate into the biliary tract due to stasis/obstruction, acute biliary tract infections can occur and induce bacteremia.

The intestinal *E. coli* population are known to express many virulence factors. Some of these factors are needed for persistent colonization of the gut, for example, *E. coli* is resistant to the effects of bile [21]. In response to bile stress, both commensal and pathogenic *E. coli* strains, and especially enteropathogenic *E. coli* strains, activate stress response pathways [22, 23], efflux pumps [24], and production of toxins [25] in the gut. As there are high concentrations of bile acid in the biliary tract, resistance against bile might play an important role in pathogenicity.

In our study, other virulence factors needed during infection, such as those related with iron acquisition, adhesion, and invasion, were analyzed.

In the present study, the *iutA* gene was found at significantly lower frequency in the *E. coli* detected in the severe group than in those of the non-severe group.

The *iutA* gene encodes the aerobactin siderophore ferric receptor protein, facilitates iron acquisition by mediating the uptake of siderophores [26]. In a chicken infection model, *iutA* expression in extraintestinal pathogenic *E. coli* strains was at least 50-fold higher compared to in vitro grown bacteria [27]. In mammalian hosts, iron is tightly bound to various proteins, such as hemoproteins and ferritin [28], making free iron availability for pathogenic bacteria scarce. In biliary tract infections, bile is an iron-limiting environment [29]. Bile stress also causes increased expression of genes encoding virulence factors associated with iron scavenging in *E. coli* [30]. Therefore, *E. coli* strains harboring the *iutA* gene may become competitive in bile, 77.3% of the *E. coli* isolates from the non-severe group and approximately 50% of those from the severe group harbored the *iutA* gene.

It has been reported that *iutA* vaccine protect mice in a sepsis challenge model [31] and urinary tract infection model [32]. This may indicate that the *iutA* gene product might be easily recognized as an antigen by host immune systems, leading to the elimination of the *E. coli* strains harboring the *iutA* gene, in bile. Paradoxically, the *iutA* gene expression constitutes both an advantage and a disadvantage for *E. coli* as permits *E. coli* proliferation in the bile, but could also increase *E. coli* susceptibility to elimination as it can be recognized by the immune system. Excessive *E. coli* proliferation might cause an increase in inflammation and the patients might decline to a more severe condition. In this study, we analyzed *E. coli* isolates from blood cultures of patients with bacteremic biliary tract infection. We hypothesized that the lower frequency of the *iutA* gene in the isolates from the severe group might be due to the elimination of the *iutA-*expressing isolates by the immune system before they translocate to the blood. Further studies will be needed to accurately explain this hypothesis, as other virulence factors different than *iutA* might be needed for translocation through the barrier between bile and blood.

In contrast, *E. coli* strains containing the *ibeA* gene were found in a higher proportion in the severe group. The *ibeA* gene encodes a 50-kDa protein that participates in *E. coli* penetration in the microvascular endothelial cells of the human brain, allowing *E. coli* to cross the blood brain barrier. This virulence factor has an important role in neonatal meningitis [33, 34]. IbeA may also be essential for the invasion of intestinal epithelial cells and macrophages [35]. The *ibeA*-positive *E. coli* were detected among adherent-invasive *E. coli* isolates from pediatric Crohn's disease patients (positive rate: 81.8%) [36], bacteremic *E. coli* isolates that translocated from the gastrointestinal tract (27%) [37] and avian pathogenic *E. coli* isolates (26%) [38].

No information regarding the relationship between the *ibeA* and biliary tract epithelium cells has been reported; therefore, the mechanism by which the *ibeA* gene expression could contribute to the severity of acute biliary infection is unknown. However, it has been reported that *E. coli* in the gut can flood into the bile duct causing acute biliary tract infection, and that *ibeA*-positive *E. coli* strains show increased oxidative stress (H_2_O_2_) resistance [39] and persistence in macrophage [35]. These characteristics might be an advantageous as allows the proliferation of *E. coli* in the bile and confers oxidative stress resistance and the possibility to escape by gathering inside the macrophages and then passing into the blood.

IbeA inhibitors have been discovered to prevent invasion of human brain microvascular endothelial cells in vitro [40]. It would be worthwhile to investigate whether *E. coli* strains harboring *ibeA* can perform internalization, and whether *ibeA* is essential to invade from bile duct to vessels through in vitro experiments using *ibeA* inhibitors.

The prevalence of the *iutA* and the *ibeA* genes and related ST lineages were evaluated in this study. All the ST131 isolates were *iutA*-positive but *ibeA*-negative. ST131 is one of the major extraintestinal pathogenic *E. coli* global lineages and ST131 clade C epidemiologically emerged from clade B, approximately in the year 2002 [41]; a comparison between clades B and C showed that all clade B isolates were *ibeA*-positive, while all clade C were *ibeA*-negative [41]. Moreover, *ibeA* gene inactivation resulted in loss of early biofilm formation [41]. In Japan, ST131 clade C emerged around the year 2003 and by 2014, all the ESBL-producing ST131 isolates were from clade C [42]. As the isolates analyzed in this study were collected from 2013 to 2015, the *ibeA* prevalence among ST131 might have been affected by the epidemiological changes of clades but the rate of the ESBL-producers in our study was just 41.2% among the ST131 isolates. The proportion of ST lineage between the severe group and the non-severe group did not show differences and the relationship between *ibeA* prevalence and the severity of bacteremia was significant.

This study has several limitations. First, although cholangitis is a polymicrobial infection [3], we have investigated only one *E. coli* strain from each patient except for one patient without analyzing other *E. coli* strains found in the bile. This comparison might reveal whether *ibeA*-positive strains can more easily escape or invade into the blood compared to strains that remained in the bile. Second, this study was conducted at a single institution; a larger multi-center study is needed to assess potential bias in the epidemiology of phylogenetic patterns and virulence factors and to be able to detect statistically significant predictors. Third, the multivariable analysis of the data including both the *iutA* and the *ibeA* genes could not be performed because the sample size was too small for the analysis to be reliable.

## Conclusion

We determined that several BEC isolates belonged to the virulent group B2 and presented diverse STs. In the *E. coli* isolates from the biliary tract of the severe group, the presence of the *iutA* gene was less frequent than in the non-severe group, while that of the *ibeA* gene was higher.

## Methods

### Patients

This retrospective study was conducted at the University of Tokyo Hospital, a 1217-bed tertiary-care teaching hospital in Tokyo, Japan. Patients with acute biliary tract infection who also had *E. coli* isolates detected in their blood were included in the study from April 2013 to February 2015. Each patient in this study was included only once, even if the patients repeatedly suffered *E. coli* bacteremia with acute biliary tract infection. Patient data, including clinical symptoms and microbiological data were collected from the medical records.

### Data collection and definitions

Patient data collected included age, sex, underlying disease (diabetes mellitus, malignancy with or without metastasis, lymphoma, and collagen disease), use of immunosuppressants, biliary tract abnormalities, such as insertion of an intrabiliary stent or surgery for biliary carcinoma, gastrointestinal tract abnormalities and past history of acute biliary infection and bacteremia. Gastrointestinal tract abnormalities were defined as stenosis of the gastrointestinal tract, and history of surgery with change of gastrointestinal route. History of residence in a nursing home and antibiotic use within three months before onset of bacteremia was also noted. Collected patient laboratory data included white blood cell counts, platelet counts, total bilirubin, aspartate aminotransferase (AST), alanine aminotransferase (ALT), alkaline phosphatase (ALP), gamma glutamyltransferase (g-GTP), and C-reactive protein (CRP). Cases in which *E. coli* was detected from blood cultures obtained within 48 h after admission were defined as community acquired infection, while cases different from those were considered hospital acquired infections.

Biliary tract infection was defined according to the Tokyo guideline [43]. Cholangitis was defined in cases where all the following criteria were positive: (1) generalized inflammation sign such as fever (more than 38.0 °C), elevation of inflammation indicators in blood tests (white blood cell counts < 4000/μL or more than 10,000/μL, and C-reactive protein 1 mg/dL or more), (2) signs of bile stasis such as jaundice (total bilirubin 2 mg/dL or more), elevation of liver function and biliary function tests (more than one-and-a-half times of the upper limit for normal values for alkaline phosphatase, gamma-glutamyltransferase, aspartate aminotransferase, or alanine aminotransferase, normal range; ALP 106–322 U/L (measured by the method of the Japan Society of Clinical Chemistry[19]), g-GTP 13–64 U/L in men and 9–32 U/L in women, AST 13–30 U/L, ALT 10–42 U/L in men and 7–23 U/L in women, respectively), and (3) imaging of biliary tract abnormalities, such as dilatation of the biliary tract or the presence of a stent, and constriction. Cholecystitis was defined as: (1) localized clinical signs such as Murphy’s sign and pain in right upper abdomen, (2) generalized inflammation such as fever and elevation of inflammation indicators in blood tests, and 3) typical findings, such as acute cholecystitis with echocardiography or CT scan. Severity was divided using the Pitt bacteremia score [44] as severe (score of 2 or more) and non-severe (score < 2).

### Microbiological procedures

All isolates were identified using the Walkaway system (Siemens, Berlin, Germany) or matrix-assisted laser desorption/ionization time-of-flight mass spectrometry (using the MALDI Biotyper; Bruker Daltonik, Germany).

The classification of the *E. coli* isolates into phylogenetic groups, such as A, B1, B2, C, D, E, and F, was performed by quadruplex polymerase chain reaction (PCR) method, as described by Clermont et al. [45]. For identification of *E. coli* sequence types (STs) 69, 73, 95, and 131, frequently detected in bacteremic isolates [46], the multilocus sequence typing PCR method [47] was used. Then, the un-typable isolates by the PCR method sequenced [48] were typed according to the EnteroBase web resource [49]. The prevalence of 20 virulence factors (*afaB* and *afaC*, *cnf1*, *cvaC*, *fimH*, *fyuA*, *hlyA*, *ibeA*, *iha*, *iroN*, *iucD*, *iutA*, *kpsMT2*, *ompT*, *papC*, *papG2*, *sat*, *sfaD* & *sfaE*, *tcpC*, *traT*, and *usp*) was screened by multiplex PCR using extracted *E. coli* genomic DNA according to previous reports [50–56].

### Statistical analysis

The two-tailed Fisher’s exact test was used for analysis of categorical data. Non-parametric data were analyzed using the Mann–Whitney *U* test. Values of *P* < 0.05 were considered significant. The variables that showed *P* < 0.05 in the virulence factor-encoding genes were entered a multivariable analysis using the multinomial logistic model. All statistical analyses were performed using JMP Pro version 11 software (SAS Institute, Cary, NC, USA).

### Ethical considerations

This study was approved by the research ethics committee at the University of Tokyo Hospital. Obtaining written informed consent from each patient was waived because it was an observational retrospective study. The data were analyzed anonymously.

## Data Availability

The datasets used and/or analyzed during the current study are available from the corresponding author on reasonable request.
